# The Cellular Functions and Molecular Mechanisms of G-Quadruplex Unwinding Helicases in Humans

**DOI:** 10.3389/fmolb.2021.783889

**Published:** 2021-11-29

**Authors:** Yang Liu, Xinting Zhu, Kejia Wang, Bo Zhang, Shuyi Qiu

**Affiliations:** ^1^ Key laboratory of Plant Resource Conservation and Germplasm Innovation in Mountainous Region (Ministry of Education), Collaborative Innovation Center for Mountain Ecology and Agro-Bioengineering (CICMEAB), College of Life Sciences/Institute of Agro-bioengineering, Guizhou University, Guiyang, China; ^2^ College of Basic Medicine, Zunyi Medical University, Zunyi, China; ^3^ The Key Laboratory of Fermentation Engineering and Biological Pharmacy of Guizhou Province, Guizhou University, Guiyang, China; ^4^ School of Liquor and Food Engineering, Guizhou University, Guiyang, China

**Keywords:** G-quadruplex, helicase, genetic disease, age-related diseases, cancer

## Abstract

G-quadruplexes (G4s) are stable non-canonical secondary structures formed by G-rich DNA or RNA sequences. They play various regulatory roles in many biological processes. It is commonly agreed that G4 unwinding helicases play key roles in G4 metabolism and function, and these processes are closely related to physiological and pathological processes. In recent years, more and more functional and mechanistic details of G4 helicases have been discovered; therefore, it is necessary to carefully sort out the current research efforts. Here, we provide a systematic summary of G4 unwinding helicases from the perspective of functions and molecular mechanisms. First, we provide a general introduction about helicases and G4s. Next, we comprehensively summarize G4 unfolding helicases in humans and their proposed cellular functions. Then, we review their study methods and molecular mechanisms. Finally, we share our perspective on further prospects. We believe this review will provide opportunities for researchers to reach the frontiers in the functions and molecular mechanisms of human G4 unwinding helicases.

## 1 Helicase

Helicases are ubiquitous motor proteins that unwind the hydrogen bonds between nucleic acids by coupling the chemical energy from nucleoside triphosphates (NTPs) (Lohman and Bjornson, 1996). The first DNA helicase was discovered and isolated from *E. coli* in 1976 by Hoffmann-Berling et al. (Abdel-Monem et al., 1976), who also first used the term ‘helicase’ in 1978. Since then, more and more helicases from all kingdoms of life have been reported (Brosh and Matson, 2020). Approximately 1% of the eukaryotic and prokaryotic genes encode helicases (Wu and Spies, 2013). Based on shared motifs, helicases can be classified into six superfamilies (SF1–6). SF1 and SF2 are two large groups and are usually monomers, whereas SF3–6 form hexamer rings (Singleton et al., 2007; Jackson et al., 2014).

Helicases are involved in almost all aspects of nucleic acid functions and metabolisms, such as DNA replication, DNA recombination, RNA transcription, translation, DNA repair, telomere maintenance, ribosome biogenesis, pre-mRNA splicing, viral RNA sensing, and miRNA biogenesis (Lohman and Bjornson, 1996; Jankowsky et al., 2011). For human beings, the first helicase was isolated from HeLa cells in 1990 (Tuteja et al., 1990), and a total of 95 non-redundant helicases including 64 RNA helicases and 31 DNA helicases were identified (Umate et al., 2011), although a few helicases can act on both RNA and DNA (Creacy et al., 2008; Talwar et al., 2017). Because of their importance, helicases are closely related to age-related diseases, such as cancer (Uchiumi et al., 2015). For example, deficiencies in RecQ family helicases BLM/RECQ2, WRN/RECQ3, and RTS/RECQ4 lead to Bloom, Werner, and Rothmund-Thomson syndromes, respectively (Croteau et al., 2014; Mojumdar, 2020). Besides the canonical duplex DNA or RNA, helicases are able to unfold other secondary structures such as Holliday junction, double Holliday junction, and G4s (Brosh, 2013).

## 2 G-Quadruplex

**FIGURE 1:**
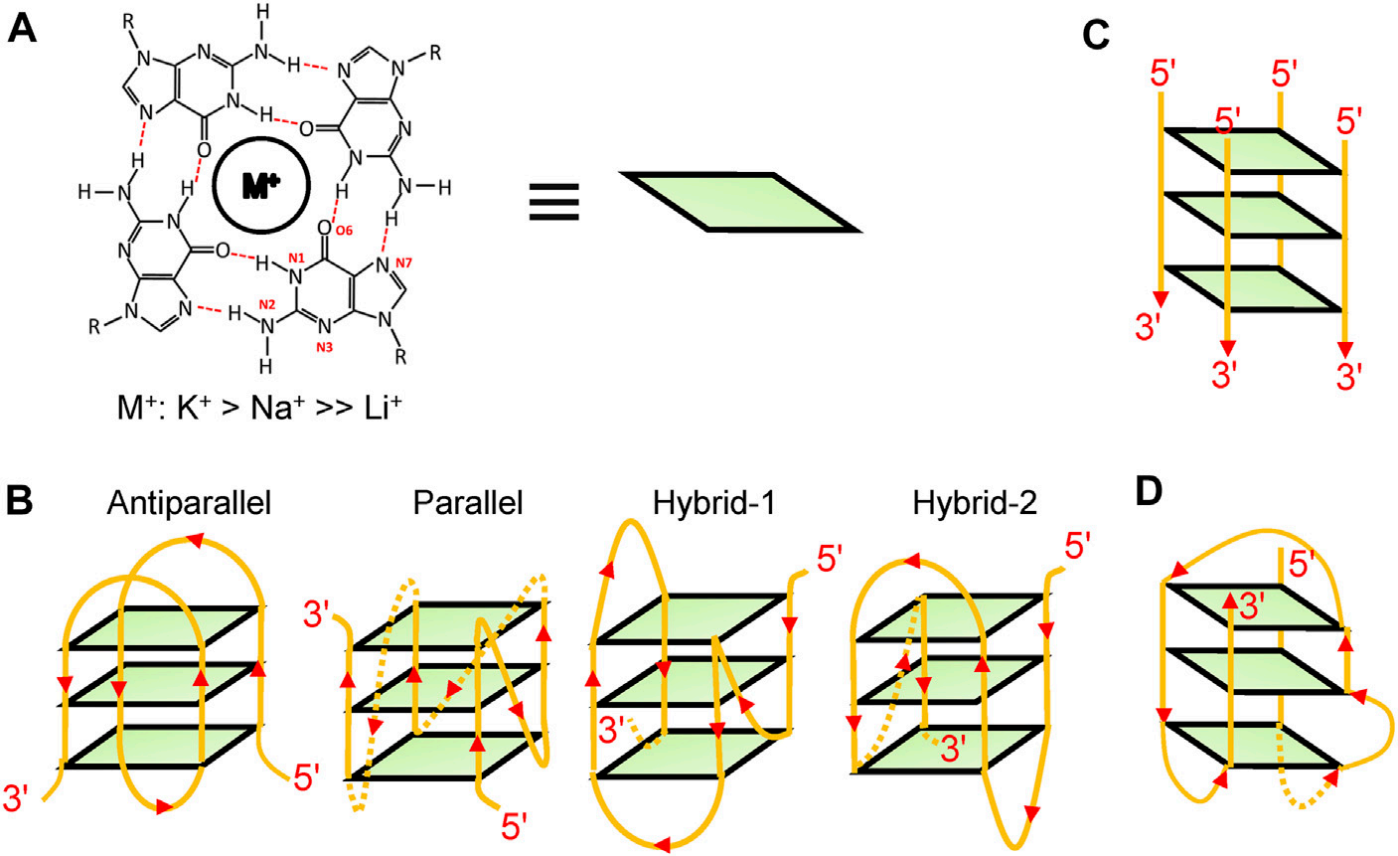
G4 structures. **(A)** A schematic of a G-quartet. **(B)** Some examples of intramolecular structures, such as parallel structures, anti-parallel structures, and hybrid structures. **(C)** A schematic of an intermolecular structure. **(D)** A bulged structure.

In living cells, guanine-rich DNA and RNA can fold into non-canonical structures called G4s, one of which is formed by stacking of at least two planar G-quartets comprising four Hoogsteen-bonded guanines and further stabilized by cations (Figure 1A) (K^+^ > Na^+^ >> Li^+^) (Lipps and Rhodes, 2009; Bhattacharyya et al., 2016). Various types of G4 structures have been reported such as intramolecular structures (Figure 1B), intermolecular structures (Figure 1C), and atypical structures (Figure 1D) (Lipps and Rhodes, 2009; Varshney et al., 2020). For intramolecular structures, diverse topological structures were identified *in vitro*, such as parallel structures, anti-parallel structures, and hybrid structures (Figure 1B). However, because of *in vivo* crowding environments, DNA G4s were considered to be more inclined to fold into parallel structures (Heddi and Phan, 2011).

**FIGURE 2:**
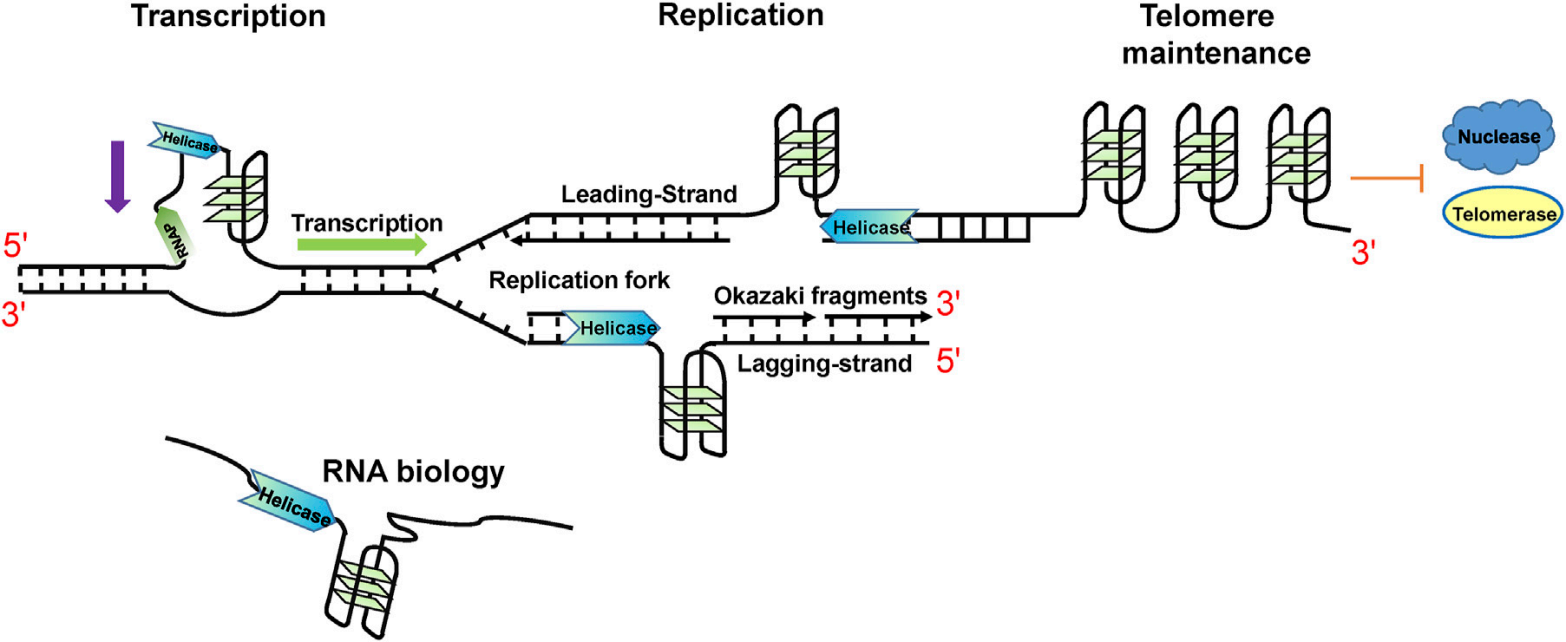
Cellular functions of specialized helicases functioning by regulating G4 structures.

G4s have been experimentally identified from multiple species (Marsico et al., 2019), from bacteria (Shao et al., 2020) to humans (Hansel-Hertsch et al., 2017), even in viruses (Saranathan and Vivekanandan, 2019). G4s have been proven to play important regulation roles. In human beings, there are about 700,000 DNA G4s identified by high-throughput G4-seq analysis (Chambers et al., 2015), and G4 structures have been shown to accumulate in the S phase within human cells (Biffi et al., 2013). In addition, by using G4 ChIP-seq with BG4 (a G4 special antibody), it was determined that approximately 10,000 DNA G4s were found mainly in regulatory and nucleosome-depleted regions (Hänsel-Hertsch et al., 2016). This suggests that most DNA G4s are found in suppressed chromatin regions, which can control the folding of G4s in cells (Shen et al., 2021). During replication and transcription, duplex DNA is unwound to ssDNA and the restriction from the complementary strand is stripped, resulting in the formation of G4 structures. This means that the emergence of G4s is strictly regulated. In addition, different cell states can influence the formation of G4s; for example, the levels of G4 structures are higher in cancer cells compared to healthy cells (Biffi et al., 2014; Hänsel-Hertsch et al., 2016). DNA G4s are not randomly distributed in the genome but are particularly enriched in telomeres, promoters, replication origins, immunoglobulin heavy chain gene switch regions, and DNA replication origins (Maizels and Gray, 2013; Tian et al., 2018; Spiegel et al., 2021). Consistent with their locations, DNA G4s are crucial in telomere maintenance, transcription, recombination, and replication (Maizels and Gray, 2013; Tian et al., 2018) (Figure 2). G4 DNA plays positive roles in telomere maintenance, replication, and transcription initiation. However, they also serve as obstacles for replication and transcription progression (as reviewed by (Lerner and Sale, 2019; Bryan, 2020; Teng F.-Y. et al., 2021; Dumas et al., 2021)).

Based on the existence of G4s in the human genome, it is not difficult to speculate that the G4 structures are also present in RNA through transcription (Figure 2). Generally, RNA G4s are more thermodynamically stable compared to DNA G4s with the same sequence (Joachimi et al., 2009). Using RNA G4-seq on mRNA from HeLa cells, more than 3,000 mRNA were found to contain G4 structures (Kwok et al., 2016) and were selectively enriched in 5′ and 3′ untranslated regions (UTRs) (Lee et al., 2020). Furthermore, RNA G4s were also reported to exist in noncoding RNAs (ncRNAs). RNA G4s not only perform important modulatory roles in RNA metabolism and function both in mRNA and ncRNA, such as pre-mRNA maturation, mRNA translocation, mRNA degradation, telomere elongation, micro RNA (miRNA) biogenesis, ribosomal RNA (rRNA) remolding, and PIWI-interacting RNA (piRNA) biogenesis, but also function in DNA metabolisms such as class switch recombination and telomere elongation (Agarwala et al., 2015; Kharel et al., 2020a; Kharel et al., 2020b). The additional 2′-OH hydroxyl groups of RNA promote intramolecular RNA G4s predominantly folding into parallel structures (Collie et al., 2010; Zhang et al., 2011), even though two antiparallel RNA G4s have been reported *in vitro* (Xiao et al., 2017; Xiao et al., 2018).

## 3 G-Quadruplex Unwinding Helicases and Their Functions

Because of their importance, G4 homeostasis in cells is precisely and dynamically regulated by interacting proteins (Brazda et al., 2014; Zyner et al., 2019; Varshney et al., 2020). If this process is impaired, it can result in serious genetic and age-related diseases (Maizels, 2015; Kharel et al., 2020a; Kharel et al., 2020b). Considering the stability of G4s and the nature of their spontaneous folding, it is particularly important for G4 to unfold properly on time. This is the reason why G4 helicases are essential (Mendoza et al., 2016; Lejault et al., 2021). For example, G4 helicases are key regulators for DNA replication and telomere lengthening. During DNA replication, dsDNA is unwound to ssDNA, and G4 structures subsequently form in both leading and lagging strands. If not resolved in a timely manner, they will threaten genome stability (Lerner and Sale, 2019). In the leading strand, the formation of G4 structures will stall replicative helicases. Some G4 helicases including FANCJ, BLM, WRN (Sarkies et al., 2012), and PIF1 (Lopes et al., 2011) were reported to unfold the G4 structures. In the lagging strand, G4s do not stall replicative helicases but their formation still requires resolution. PIF1 (Dahan et al., 2018), and FANCJ (Sato et al., 2021) were reported to unfold lagging strand G4s. During telomere dsDNA replication, WRN was found to unfold G4s in lagging strand (Crabbe et al., 2004). In addition, BLM and FANCJ was observed to unfold G4s in leading strand (Drosopoulos et al., 2015). During alternative lengthening of telomere (ALT), telomeric G4s serve as barriers and should be unfolded (Clynes et al., 2015). RECQ1 may play a role in this process (Popuri et al., 2014). The 3′ ssDNA G-rich tail can fold into G4s, proposed to play role in capping telomeres. Telomerase can elongate this 3’ tail directly with the help of G4 unwinding helicases such as BLM (Wang et al., 2011). Furthermore, some helicases, such as RTEL1 (Ghisays et al., 2021) and DHX36 (Booy et al., 2012), can maintain telomeres through interaction with RNA G4s. It is clear that not all helicases can efficiently unfold G4 (Popuri et al., 2008; Bharti et al., 2014; Zhou et al., 2014; Wu et al., 2020), and at least 24 human helicases belonging to SF1 and SF2 have been reported to be associated with G4s, as shown in Table 1. Presently, only SF1 and SF2 helicases are reported to unwind G4 structures in humans, even though SV40 T-antigen from the SV40 virus belonging to SF3 was reported to unwind G4 structures *in vitro* (Baran et al., 1997). The reason why there are few SF3-6 helicases reported to unfold G4s may be that most ring type SF3-6 helicases function as the primary helicases in chromosomal DNA replication (Lohman et al., 2008), during which they are blocked by G4 structures (Lerner and Sale, 2019).

**TABLE 1:** Human helicases have been proposed to bind and/or unwind G4s.

SF	Family	Helicase	DNA or RNA	Disease	Direction	G4-modulated functions
SF1		PIF1	DNA G4 Sanders, (2010)	Cancer predisposition Chisholm et al. (2012)	5′-3′	Telomere maintenance and DNA replication Paeschke et al. (2013)
		DNA2	DNA G4 Masuda-Sasa et al. (2008)		5′-3′	Telomere maintenance and DNA replication Masuda-Sasa et al. (2008)
		MOV10	RNA G4 Zhang et al. (2019c)		5′-3′	Translation regulation Kenny et al. (2014)
		MOV10L1	RNA G4 Zhang et al. (2019c)		5′-3′	piRNA biogenesis and function Vourekas et al. (2015)
SF2	RecQ	RECQ1	DNA G4 Liu et al. (2021b)		3′-5′	Telomere maintenance Popuri et al. (2014) and transcription Li et al. (2014), Lu et al. (2016)
		BLM	DNA G4 Sun et al. (1998); Wu et al. (2015)	Bloom syndrome Cheok et al. (2005)	3′-5′	Telomere maintenance Drosopoulos et al. (2015), replication Drosopoulos et al. (2015), van Wietmarschen et al. (2018), recombination van Wietmarschen et al. (2018), and transcription Nguyen et al. (2014)
		WRN	DNA G4 Fry and Loeb, (1999); Wu et al. (2017)	Werner syndrome Shen et al. (1998)	3′-5′	Telomere maintenance Crabbe et al. (2004), replication Crabbe et al. (2004); Sarkies et al. (2012), and transcription Tang et al. (2016)
		RECQ5β	DNA G4 Budhathoki et al., (2016)	Cancer predisposition Peng et al. (2019)	3′-5′	
	Fe-S	FANCJ	DNA G4 London et al. (2008); Bharti et al. (2013)	Fanconi anemia Wu et al. (2009)	5′-3′	Replication Lee et al. (2021)
		DDX11/ChlR1	DNA G4 Wu et al. (2012)	Warsaw breakage syndrome Wu et al. (2012)	5′-3′	Replication van Schie et al. (2020)
		XPD	DNA G4 Gray et al. (2014)	Xeroderma pigmentosum Hanaoka, (2013)	5′-3′	Transcription Gray et al. (2014)
		RTEL1	DNA G4 Vannier et al. (2013)	Hoyeraal-Hreidarsson syndrome Le Guen et al. (2013)	5′-3′	Telomere maintenance Vannier et al. (2012), Vannier et al. (2013), replication Kotsantis et al. (2020), and transcription Kotsantis et al. (2020)
	DEAD	DDX1	RNA G4 Ribeiro de Almeida et al. (2018) and DNA G4 Zhang et al. (2021a)			IgH class switch recombination Ribeiro de Almeida et al. (2018)
		DDX2/eIF4A	RNA G4 Wolfe et al. (2014)	Cancer Wolfe et al. (2014)		Translation regulation Wolfe et al. (2014)
		DDX3X	RNA G4 Herdy et al. (2018)			Transcription Herdy et al. (2018) and rRNA remolding Mestre-Fos et al. (2019)
		DDX5	RNA G4 Dardenne et al. (2014), Herdy et al. (2018) and DNA G4 Wu et al. (2019)			Transcription Herdy et al. (2018); Wu et al. (2019) and pre-mRNA splicing Dardenne et al. (2014)
		DDX17	RNA G4 Dardenne et al. (2014), Herdy et al. (2018)			Transcription Herdy et al. (2018), pre-mRNA splicing Dardenne et al. (2014), and rRNA remolding Mestre-Fos et al. (2019)
		DDX21	RNA G4 McRae et al. (2017)			Translation regulation McRae et al. (2017); McRae et al. (2020) and rRNA remolding Mestre-Fos et al. (2019)
		DDX24	DNA G4 Zhang et al. (2021a)			
		DDX42	DNA G4 Zyner et al. (2019)			
		DDX58	RNA G4 Herviou et al. (2020)			
		DEAH	DHX9/RNA helicase A	RNA G4 Chakraborty and Grosse, (2011) and DNA G4 Chakraborty and Grosse, (2011)		3′-5′	Translation regulation Murat et al. (2018)
			DHX36/RHAU	RNA G4 Creacy et al. (2008) and DNA G4 Creacy et al. (2008), Chen et al. (2015), Tippana et al. (2016), You et al. (2017)		3′-5′	Translation regulation Murat et al. (2018), pre-mRNA polyadenylation Newman et al. (2017), mRNA localization Maltby et al. (2020), mRNA degradation Tran et al. (2004), telomere regulation Booy et al. (2012), lncRNA function Booy et al. (2016), miRNA function Booy et al. (2014), and transcription Huang et al. (2011)
		Snf2	ATRX	DNA G4 Law et al. (2010)	ATRX syndrome Gibbons et al. (1995)		Telomere maintenance Nguyen et al. (2017), replication Wang et al. (2019); Teng et al. (2021b), and transcription Levy et al. (2014)

### Superfamily 1

Petite integration factor 1 (PIF1) helicases are 5′-3′ DNA helicases, which are widely present in prokaryotic to eukaryotic organisms, and are outstanding for their potent G4 unwinding activity (Ribeyre et al., 2009; Paeschke et al., 2013; Liu et al., 2015; Xue et al., 2020). For eukaryotes, the cellular functions of PIF1 have been elucidated through yeast studies. Both ScPIF1 from *Saccharomyces cerevisiae* and Pfh1 from *Saccharomyces pombe* were shown to bind and unwind G4 structures (Byrd and Raney, 2015; Wallgren et al., 2016) and promote DNA replication at G4s to prevent DNA damage (Ribeyre et al., 2009; Paeschke et al., 2011; Sabouri et al., 2014). In addition, these two helicases also play essential roles in telomere maintenance. SpPIF1 (Pfh1 from *S. pombe*, fission yeast) facilitates telomere replication, and ScPIF1 (from *S. cerevisiae*, budding yeast) is a negative regulator of telomerase which prevents telomere lengthening. In *S. cerevisiae*, the PIF1 family helicase ScRrm3 promotes telomere replication but does not affect telomerase (Ivessa et al., 2002; Paeschke et al., 2013). Human PIF1 can also resolve G4 DNA *in vitro* (Sanders, 2010) and suppress G4-induced DNA damage and telomere lengthening (Mateyak and Zakian, 2006; Paeschke et al., 2013). Though human PIF1 was thought to play an important role in reassembling stalled DNA replication forks (George et al., 2009), there is no direct evidence that it is involved in resolving G4 structures in this process. Furthermore, PIF1 defects in patients result in cancer predisposition (Chisholm et al., 2012).

Human DNA2 helicase can bind and unfold G4 structures, with this activity predicted to occur in DNA replication (Okazaki fragment processing) and telomere elongation (Masuda-Sasa et al., 2008). In mouse models, DNA2 can protect the integrity of telomeres by cleaving G4 DNA (Lin et al., 2013).

Another two human SF1 helicases, MOV10 (moloney leukemia virus 10) and its paralog moloney leukemia virus 10 like 1 (MOV10L1), are associated with different RNA regulatory pathways. Both are preferable to bind G4 RNA *in vivo* (Kenny et al., 2014; Vourekas et al., 2015). MOV10 binding G4 RNA is involved in fragile X mental retardation protein (FMRP)-mediated translational regulation (Kenny et al., 2014), and MOV10L1 binding G4 RNA is associated with piRNA biogenesis and function (Vourekas et al., 2015). Both MOV10 and MOV10L1 can unwind RNA G4 *in vitro*, and the unwinding activity of MOV10L1 is more efficient than that of MOV10 (Zhang X. et al., 2019).

### Superfamily 2

#### 3.1.1 RecQ Family

The RecQ family is one of the most studied G4 unwinding families with 3′-5′ translocation direction. *E. coli* contains one RecQ helicase, while *S. cerevisiae* has two, namely, Sgs1, and Hrq1, all of which have efficient G4 DNA unwinding activity (Sun et al., 1999; Wu and Maizels, 2001; Rogers et al., 2017). Recently, Plant RecQ helicase AtRECQ2 was identified to be able to efficiently unfold plant telomeric G4, but AtRECQ3 cannot (Wu et al., 2020). Humans and most mammals have five RecQ helicases: RECQ1, BLM/RECQ2, WRN/RECQ3, RTS/RECQ4, and RECQ5 (Croteau et al., 2014). Three of them, namely, BLM, WRN, and RECQ5, have G4 unwinding activity (Fry and Loeb, 1999; Croteau et al., 2014; Drosopoulos et al., 2015; Wu et al., 2015; Budhathoki et al., 2016; Wu et al., 2017).

Even RECQ1 was reported to inefficiently unfold intermolecular G4s *in vitro* (Popuri et al., 2008). However, most recently, Xi’s lab reported that the unwinding activity of *Bos taurus* RECQ1 was modulated by its oligomeric states. In addition, both monomeric BtRECQ1 and monomeric human RECQ1 unfolded intramolecular G4 DNA as efficiently as human BLM helicase (Liu N.-N. et al., 2021). There are evidences suggest that RECQ1 can function through interaction with G4s. For example, RECQ1 is able to bind G4s in the promoter region of genes that will be downregulated when RECQ1 is silenced (Li et al., 2014; Lu et al., 2016). Additionally, RECQ1 is also involved in telomere maintenance in alternative lengthening of telomere (ALT) cells, most likely in the process of telomere replication (Popuri et al., 2014), when telomeric G4 acting as a barrier should be unfolded (Clynes et al., 2015). RECQ4 does not have a traditional integrated RecQ C-terminal (RQC) domain, which was proven to recognize G4 structure (Huber et al., 2006; Croteau et al., 2014); thus, RECQ4 is not supposed to efficiently unfold G4 structures (Croteau et al., 2014), though its N-terminus bound G4 DNA tightly (Keller et al., 2014). Additionally, RecQ4 was even capable of competing with BLM to protect G4 DNA from unfolding by BLM (Singh et al., 2012).

BLM helicase was first identified from its mutation that causes Bloom syndrome. Bloom syndrome is characterized by dwarfism, genetic instability, and cancer predisposition (Karow et al., 1997; Cheok et al., 2005). BLM can unwind a wide range of DNA structures and was one of the first identified G4 unfolding helicases (Sun et al., 1998). It exhibits particular activity in both intermolecular and intramolecular G4 DNA in an ATP-dependent manner (Sun et al., 1998; Wu et al., 2015). The 3’ ssDNA overhang was found to be indispensable for BLM loading and resolving G4 structures (Sun et al., 1998; Wu et al., 2015), and the G4 DNA hindered downstream duplex unwinding (Chatterjee et al., 2014; Wu et al., 2015). In cells, BLM was reported to play crucial roles in G4 DNA associated processes, such as telomere maintenance (Drosopoulos et al., 2015), replication (Drosopoulos et al., 2015; van Wietmarschen et al., 2018), recombination (van Wietmarschen et al., 2018), and transcription (Nguyen et al., 2014). BLM facilitates telomere replication on the leading strand by unwinding G4 structures (Drosopoulos et al., 2015) and also resolves G4 in internal genomic regions to smooth replication (Drosopoulos et al., 2015; van Wietmarschen et al., 2018). BLM helicase is able to suppress recombination at G4 structures, the accumulation of which may be accelerated by transcription (van Wietmarschen et al., 2018). BLM also modulates gene expression, which may contribute to the pathogenesis of Bloom syndrome by unwinding G4s at transcription start sites (Nguyen et al., 2014).

WRN helicase mutations result in Werner syndrome, characterized by premature aging and cancer prediction (Shen et al., 1998). It has robust G4 DNA unwinding activity *in vitro* in an ATP-dependent manner (Fry and Loeb, 1999; Wu et al., 2017). Without WRN in human cells to unfold telomere G4 structures, the lagging-strand of telomere replication is impaired (Crabbe et al., 2004). It can also unfold G4s to promote genome replication together with fanconi anemia complementation group J (FANCJ) (Sarkies et al., 2012). WRN modulates gene transcription in a G4-dependent manner, and the modulated genes are speculated to be related to Werner syndrome (Johnson et al., 2009; Tang et al., 2016). WRN interacts with BLM (von Kobbe et al., 2002), and the BLM and WRN complex assembly are facilitated by HECT and RLD domain containing E3 ubiquitin protein ligase 2 (HERC2), which suppress G4 accumulation *in vivo* (Wu et al., 2018).

Humans contain three isoforms of RECQ5 from alternative splicing, namely, α, β, and γ. Among them, only RECQ5β has helicase and ATPase activities (Ren et al., 2008). Compared to BLM and WRN, the G4 DNA unwinding activity of RECQ5β is relatively weak *in vitro* (Budhathoki et al., 2016), and there is no evidence that it modulates G4 structures to regulate cellular processes in cells.

#### 3.1.2 Fe-S Family

The hallmark of Fe-S family helicases is their Fe-S domain, which is proposed to serve as a part of a wedge for DNA unwinding with 5′-3′ unwinding direction (Estep and Brosh, 2018). Fe-S family helicases, including FANCJ, DDX11/ChlR1, xeroderma pigmentosum complementation group D (XPD), and regulator of telomere elongation helicase 1 (RTEL1), are closely related to G4 function modulation (White, 2009; Guo et al., 2015; Estep and Brosh, 2018).

The first Fe-S helicase identified as the G4-unwinding helicase in cells is DOG-1, the homologue of FANCJ from *Caenorhabditis elegans* (Cheung et al., 2002). Its mutation causes the accumulation of G4 motifs that threaten genome stability. Direct evidence using *Xenopus* egg extracts have shown that FANCJ helicase promotes DNA replication through G4 structures (Castillo Bosch et al., 2014). Human FANCJ can unwind G4 DNA *in vitro* with 5′-3′ directionality (London et al., 2008; Wu and Spies, 2016), and *in vivo*, it maintains genomic stability at G4 DNA through two independent ways: one is by coupling with REV1 polymerase, and the other is by being assisted by WRN or BLM (3′-5′ unwinding) (Sarkies et al., 2012; Estep and Brosh, 2018). Recently, FANCJ’s interaction with replication protein A (RPA) to unwind G4s at a replication fork was monitored by single-molecule imaging (Lee et al., 2021). It was believed to be the key G4 unwinding helicase during DNA replication (Brosh and Wu, 2021). Human FANCJ is essential for human health, because its mutations are closely related to Fanconi anemia and breast cancer (Wu et al., 2009), and as expected, FANCJ patient cells accumulate deletions near G4-forming sequences (London et al., 2008).

Mutations in DDX11/ChlR1 cause Warsaw Breakage syndrome (Wu et al., 2012). DDX11 was reported to bind and unfold G4 DNA *in vitro* (Wu et al., 2012) and *in vivo* during replication to prevent DNA damage (Lerner et al., 2020). Recently, it was reported that DDX11 defective cells were more sensitive to G4-stabilization than FANCJ defective cells, with the helicase domain of DDX11 being indispensable in response to G4 stabilizers. Therefore, DDX11 was proposed to resolve G4 structures, which promotes normal replication (van Schie et al., 2020).

XPB and XPD are 2 of the 11 subunits in the transcription complex TFIIH, which functions in transcription initiation (Egly and Coin, 2011). They are absolutely essential in humans, and their mutation will result in xeroderma pigmentosum (Hanaoka, 2013). Both XPB and XPD can bind G4 DNA; however, only XPD can efficiently unfold G4 DNA (Gray et al., 2014). By ChIP-seq analysis, 40% of XPB and XPD binding sites were found to overlap with G4 motifs, and the modulated genes are linked to specific cancers (Gray et al., 2014).

RTEL1, a DEAH helicase, can unwind telomeric G4s *in vitro* (Vannier et al., 2013) and is involved in telomere-length regulation by counteracting G4 DNA (Vannier et al., 2012; Vannier et al., 2013). When lacking RTEL1, cells exhibit short and fragile telomeres, which is the cause of Hoyeraal–Hreidarsson syndrome (Le Guen et al., 2013). In addition, RTEL1-deficient cells lose single-stranded G-rich overhangs (Porreca et al., 2018), which are proposed to form G4 structures. RTEL1 can also modulate replication-transcription collisions by unwinding G4s induced by R-loop (Kotsantis et al., 2020). Recently, RTEL1 was shown to function in G4 RNA. It influenced the abundance and location of telomere repeat containing RNAs (TERRAs) through binding G4 structures, but not TERRA sequences (Ghisays et al., 2021).

#### 3.1.3 DEAD and DEAH Families

RNA helicases play essential roles in RNA metabolism. RNA G4s may participate in disease pathogenesis through the regulation of mRNA and ncRNA metabolisms. For mRNA, these processes include mRNA maturation, alternative splicing, mRNA translocation, translation, and noncanonical translation for mRNA. In terms of ncRNA, G4s can play a role in telomere-associated RNAs, long noncoding RNAs, tRNA-derived stress-induced RNAs, mitochondrial RNAs, microRNAs, PIWI-interacting RNAs, and even rRNA (as discussed by (Cammas and Millevoi, 2016; Kharel et al., 2020a)). The roles of specialized DEAD and DEAH helicases regulating these processes through G4 RNA are listed in Table 1.

Most RNA helicases belong to DEAD, DEAH, and DExH protein families (Owttrim, 2013). Some helicases belonging to DEAD and DEAH families have been identified as RNA G4 unwinding helicases (Table 1). DEAD family helicases are typical poorly (or non) processive and bidirectional unwinding helicases (Cordin et al., 2006). However, DEAH family helicases usually unwind in a polarized manner. In addition, some of these helicases also modulate G4 DNA (Table 1). Compared with G4 DNA specialized helicases, the identification of G4 RNA helicases is relatively late and is still considered an emerging topic. Thus, the biochemical and biophysical characterizations of G4 RNA helicases still need to be studied.

DDX1 can bind and unfold RNA G4 both *in vitro* and *in vivo* to modulate IgH class switch recombination (Ribeiro de Almeida et al., 2018). DDX2/eIF4A is well known as a eukaryotic translation initiation factor for unfolding secondary RNA structures in the 5′-UTR to promote translation. G4 RNA is also enriched in 5′-UTR and can be regulated by eIF4A, and this process is directly related to cancer development [83]. DDX3X was also reported to unfold 5′-UTR G4 RNA of the NRAS oncogene (Herdy et al., 2018). Recently, DDX3X was speculated to perform rRNA remolding by directly modulating rRNA G4 (Mestre-Fos et al., 2019). DDX5 and DDX17 can function in transcription through interacting with G4 RNA (Herdy et al., 2018) and in pre-mRNA splicing by cooperating with heterogeneous nuclear ribonucleoprotein (hnRNP) H/F at G4 RNA (Dardenne et al., 2014). Besides these processes, DDX5 can also unwind Myc promoter DNA G4 to facilitate its transcription (Wu et al., 2019), and DDX17 may also modulate rRNA G4 just as DDX3X (Mestre-Fos et al., 2019). Furthermore, DDX21, an RNA G4 helicase (McRae et al., 2017), was also found to directly bind rRNA G4s (Mestre-Fos et al., 2019) to modulate rRNA functions. It can also resolve the 5′-UTR G4 RNA to regulate MAGE family member D2 (MAGED2) protein level (McRae et al., 2020) and the 3′-UTR G4 RNA to suppress gene expression (McRae et al., 2017). Most recently, DDX1, DDX24, DDX42, and DDX58 were identified to bind G4 DNA or RNA; however, the cellular functions are still elusive (Zyner et al., 2019; Herviou et al., 2020; Zhang X. et al., 2021).

DHX9/RNA helicase A can unfold both RNA and DNA G4s *in vitro* (Chakraborty and Grosse, 2011) and promote translation by resolving 5′-UTR RNA G4s (Murat et al., 2018). RNA helicase associated with AU-rich element (RHAU), also known as DHX36 or G4R1 was identified as the key to unwinding G4 in HeLa cell lysates (Vaughn et al., 2005) and can efficiently resolve both DNA and RNA G4s *in vitro* (Creacy et al., 2008; Chen et al., 2015; Tippana et al., 2016; You et al., 2017), even with very stable hexanucleotide repeat RNA G4 (Liu H. et al., 2021). Among RNA G4 unfolding helicases, DHX36 was thought to unwind most RNA G4s (Creacy et al., 2008; Chen et al., 2018d). As RNA helicase, DHX36 functions in the following processes: translation regulation (Murat et al., 2018; Chen et al., 2021), pre-mRNA polyadenylation (Newman et al., 2017), mRNA localization (Maltby et al., 2020), mRNA degradation (Tran et al., 2004), telomere regulation (Booy et al., 2012), lncRNA function (Booy et al., 2016), and miRNA function (Booy et al., 2014). Interestingly, although the YY1 gene contains DNA G4 in its promoter and RNA G4 in the 5′ UTR of its mRNA, DHX36 functions only on DNA G4 (Huang et al., 2011).

#### 3.1.4 SNF2 Family

Alpha-thalassemia mental retardation X-linked protein (ATRX) is an SWI2/SNF2 DNA helicase, and the decrease of its activity or expression causes ATRX syndrome (Gibbons et al., 1995). ATRX can bind G4 DNA (Law et al., 2010; Zyner et al., 2019) but cannot unwind G4 DNA since SWI2/SNF2 family helicases do not have helicase activity (Thomä et al., 2005). ATRX prevents DNA G4 formation within R-loops to prevent replication stalling and maintain telomeres (Nguyen et al., 2017). In isogenic glioma model systems, defects in ATRX lead to G4-related replication stress and DNA damage (Wang et al., 2019; Teng Y.-C. et al., 2021), implying its function during DNA replication. ATRX can also promote gene expression through G4 regions (Levy et al., 2014).

## 4 Representative Research Methods to Reveal Molecular Mechanisms

To understand the physiological functions of G4 unwinding helicases, dissecting their molecular mechanisms is fundamentally important. The understanding of the G4 unwinding mechanism is inseparable from the progress of methods. Representative assays for measuring helicase-catalyzed G4 unwinding mainly include electrophoresis, real-time fluorescence methods, single-molecule methods, and structural methods according to the chronological order and the degree of refinement.

### 4.1 Research Methods

#### 4.1.1 Electrophoresis

**FIGURE 3:**
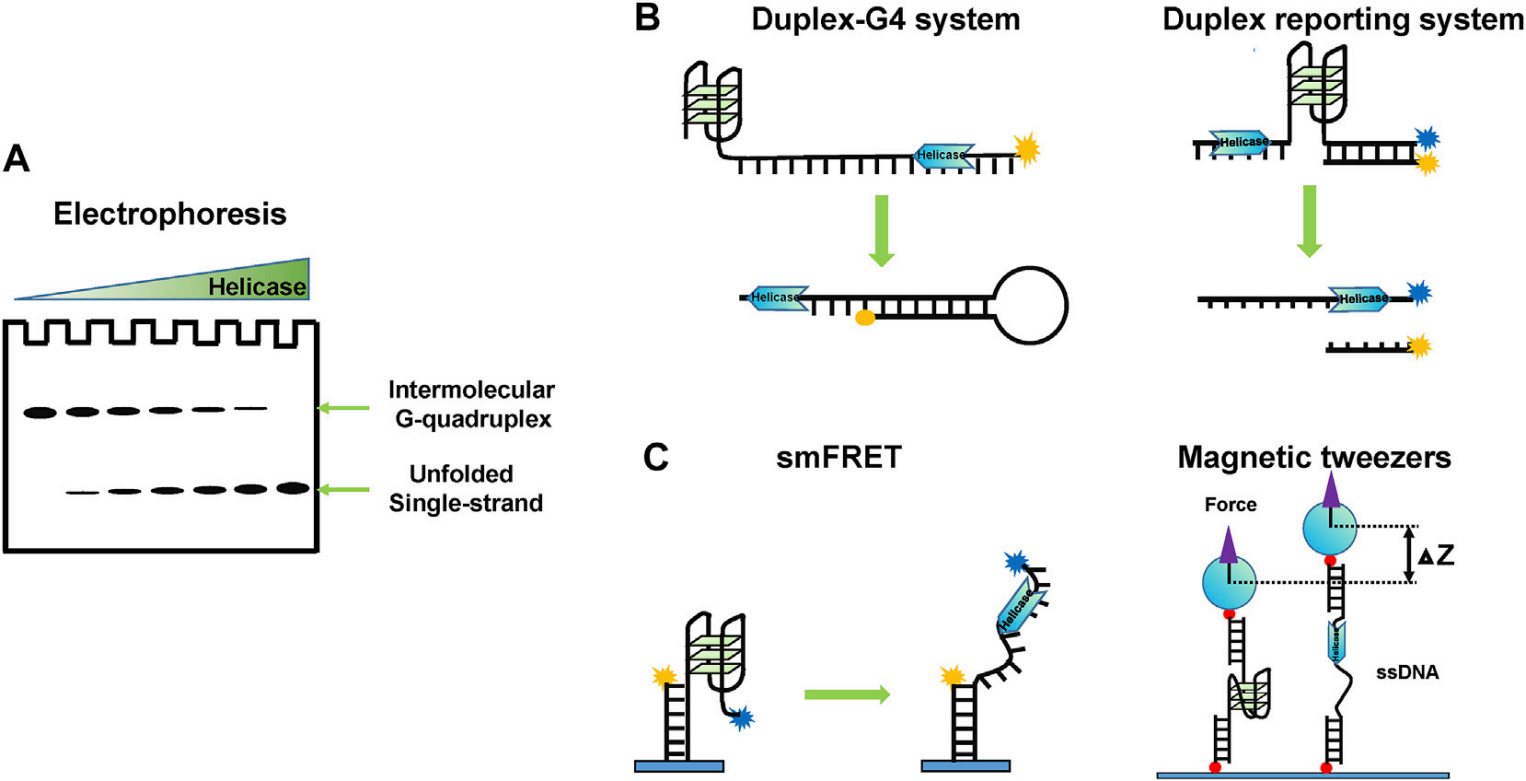
Schematic representation of representative research methods for studying G4 unwinding helicase. **(A)** Electrophoresis based on gel mobility shift. **(B)** Real-time fluorescence methods using an intramolecular trap or a duplex reporting system. **(C)** Single-molecule methods, such as smFRET and magnetic tweezers.

In the early stages, people used traditional gel mobility shift to study whether a specific helicase can unwind G4 structures, and the G4s used usually are intermolecular (Figure 3A). This method is visualized and easy to compare to the unwinding of other substrates quantificationally. Using this method, the G4 unwinding ability of many helicases, including PIF1 (Sanders, 2010), DNA2 (Masuda-Sasa et al., 2008), BLM (Sun et al., 1998), and WRN (Fry and Loeb, 1999), has been observed. Using a trap oligonucleotide, intramolecular G4s can also be analyzed by gels, and this is especially suitable for RNA G4 unwinding experiments (Zhang X. et al., 2019), since RNA G4s are usually intramolecular. However, it is not suited for micromechanism studies.

#### 4.1.2 Real-Time Fluorescence Methods

Real-time fluorescence methods are useful for monitoring the kinetic process of G4 unwinding. Tan’s lab designed a duplex-G4 system, where a complementary ssDNA tail is annealed to prevent the refolding of unwound G4 DNA (Liu et al., 2010) (Figure 3B, left panel). Recently, Xi’s lab developed a fluorescence resonance energy transfer (FRET)-based system to detect helicases unwinding G4 DNA using stopped-flow technique in real-time (Liu et al., 2020). The unwinding of the double-stranded DNA downstream of G4 can also be used to report the unwinding of G4s indirectly (Figure 3B, right panel) (Duan et al., 2015; Mendoza et al., 2015). However, the fluorescence intensity does not always reflect the unwinding fraction because the fluorescence intensity can be affected by helicases (Fischer et al., 2012).

#### 4.1.3 Single-Molecule Methods

There is no doubt that single-molecule methods have revolutionized our ability to directly investigate G4 unwinding mechanisms (Figure 3C). Using single-molecule methods, the interaction of G4s with helicases can be monitored in real-time and unwinding intermediate states can be captured directly. Ha’s lab and Xi’s lab first used single-molecule FRET (smFRET) to study ScPIF1 unfolding G4 DNA structures (Zhou et al., 2014; Hou et al., 2015), following which more and more molecular mechanisms of G4 DNA helicases have been revealed, such as BLM (Chatterjee et al., 2014; Wu et al., 2015), WRN (Tippana et al., 2016; Wu et al., 2017), FANCJ (Wu and Spies, 2016), and DHX36 (Tippana et al., 2016; You et al., 2017). For RNA G4, only DHX36 was studied by smFRET (Tippana et al., 2019; Liu H. et al., 2021). However, smFRET cannot capture atomic-level interactions, which require the intervention of structural methods.

#### 4.1.4 Structures

**FIGURE 4:**
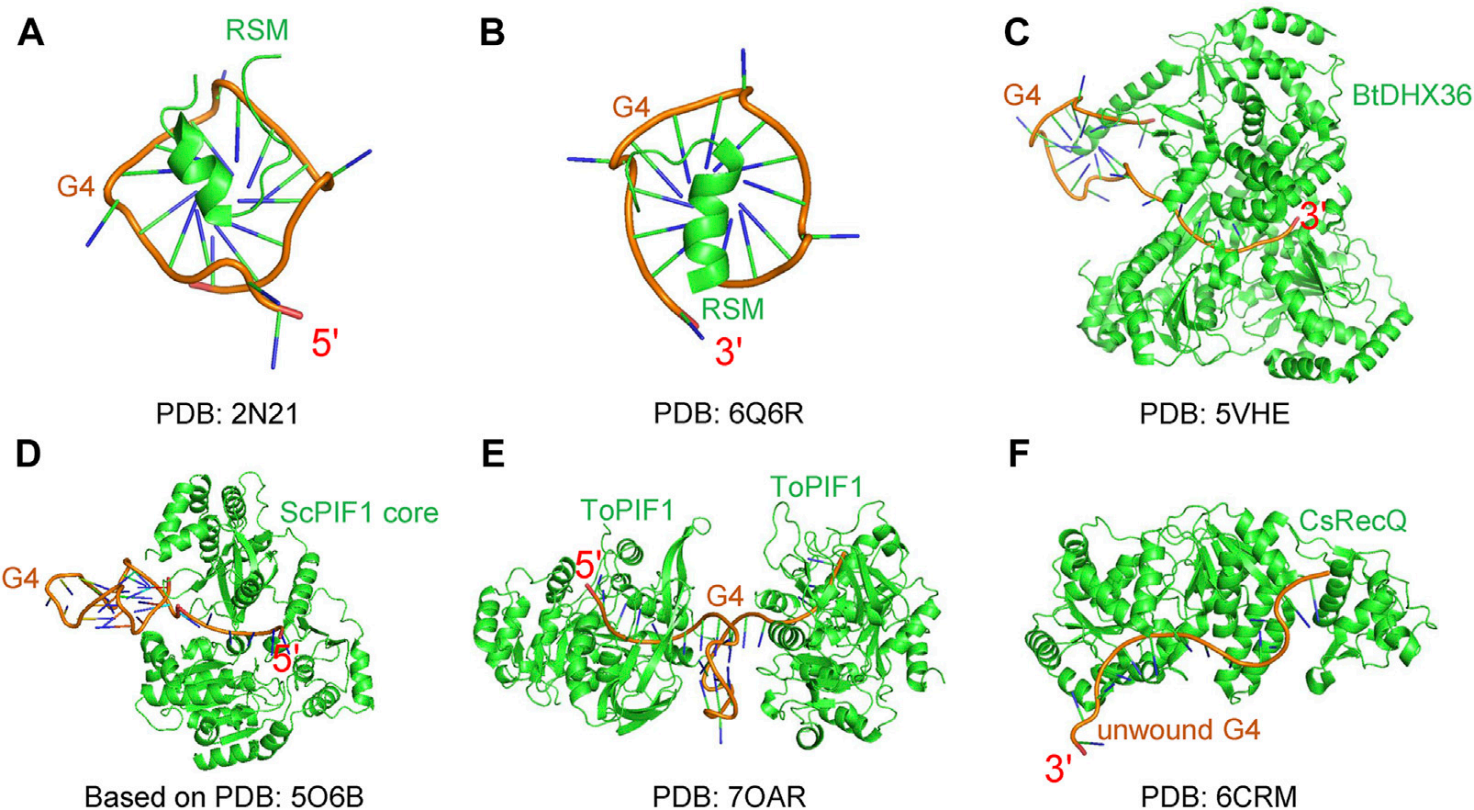
G4 DNA and protein complexes. **(A–C)** DHX36 related complexes include RSM binding 5′ G4 DNA **(A)**, RSM binding 3′ G4 DNA **(B)**, and Bos taurus DHX36-Myc G4 **(C)**. **(D)** The model of ScPIF1 core and G4 complex based on SAXS data. **(E)** *Thermus oshimai* PIF1-G4 complex. **(F)** *Cronobacter sakazakii* RecQ and unfolded G4 complex.

Structural methods can capture the detailed helicase-G4 interactions at the atomic level. There are some structural works attempting to obtain helicase-G4 structure complexes (Figure 4) (Chen W.-F. et al., 2018; Lu et al., 2018; Voter et al., 2018); however, because G4 structures are dynamic, it is not easy to obtain the complex structures. Among one of the best studied G4 unwinding helicases, using structural biology methods, is DHX36. Anh Tuân Phan’s group first resolved the 18 amino acid RHAU-specific motif (RSM) binding to 5′ parallel G4 DNA by nuclear magnetic resonance (NMR) (Figure 4A) (Heddi et al., 2015). They then obtained the crystal complex of RSM and 3′ parallel G4 DNA (Figure 4B) (Heddi et al., 2020). The crystal complex of *Bos taurus* DHX36-Myc G4 was the first real helicase-G4 structure in which G4 was also recognized by RSM, and showed a pulling mechanism (Figure 4C) (Chen M. C. et al., 2018). In addition, PIF1-G4 and RecQ-G4 complex structures also made some important progress. Based on structural analyses, small-angle X-ray scattering (SAXS) data, modeling and mutational studies, ScPIF1 core was thought to use its positively charged residues to recognize G4 DNA (Figure 4D) (Lu et al., 2017). Most recently, the *Thermus oshimai* PIF1-G4 complex was submited, showing a G4-recognizing surface (Figure 4E) (Dai et al., 2021), which is different from DHX36. In terms of RecQ family helicases, the RQC interaction with G4 DNA was studied by NMR (Lee et al., 2019), and the crystal structure of a *Cronobacter sakazakii* RecQ, bound to unfolded G4 DNA, was achived (Figure 4F), showing that a guanine-specific pocket is essential for G4 resolution (Voter et al., 2018). Interestingly, the G4s in these complexes are parallel structures with short loop lengths (Bugaut and Balasubramanian, 2008), which have higher melting temperature. This explains why they can form stable complexes with the proteins. Even though structural methods provide the clearest interaction details, these methods lose the kinetic processes of the reaction. The intermediate states from single-molecule methods are obtained by calculation and inference, and are not directly seen. Directly capturing the intermediate states through structural methods is the primary problem encountered at present. In addition, the unwinding mechanisms *in vivo* are also worth studying and are the ultimate goal of researchers.

### 4.2 Unwinding Mechanisms

For SF1, PIF1 family helicases are among the most studied. A monomeric ScPIF1 can sequentially and repetitively unfold G4 DNA (Hou et al., 2015), or anchor at 3′-tailed DNA junctions to reel in the 3′ tail ssDNA and repetitively unfold G4s step by step (Zhou et al., 2014). However, monomeric ScPIF1 cannot efficently unfold dsDNA (Zhou et al., 2014). ScPIF1 dimerization is needed for G4 downstream dsDNA unwinding, and G4/G-rich sequences can stimulate this process (Duan et al., 2015; Zhang et al., 2016). Additionally, ScPIF1 unfolds antiparallel G4s more efficiently than parallel G4s having similar stability (Byrd and Raney, 2015; Wang et al., 2018). MOV10 preferentially binds 5′ ssRNA tails and translocates on ssRNA to unwind RNA G4s. However, MOV10L1 binds ssRNA-G4 junctions more tightly and can unfold RNA G4s directly (Zhang X. et al., 2019).

In terms of SF2, the mechanisms of RecQ family helicases have been revealed. The recognition domain of RecQ is positioned in RQC (Huber et al., 2006; Croteau et al., 2014), and the helicase and RNaseD C-terminal (HRDC) domain is also essential for G4 unwinding (Chatterjee et al., 2014; Teng et al., 2020; Wu et al., 2020). BLM can translocate to unwind 3′ ssRNA-G4, and can alternatively anchor at the ssDNA-dsDNA junction to reel and unfold G4 DNA repetitively with intermediates including hairpin and G-triplex (Wu et al., 2015), similar to ScPIF1 (Zhou et al., 2014). These two unwinding manners are conserved for WRN (Wu et al., 2017). However, WRN can reciprocate on G4 structures from translocation and sliding back, driven by ATP and G4 spontaneous folding respectively (Wu et al., 2017). For Fe-S family, FANCJ can directly contact with G4 DNA via lysine residues K141 and/or K142, and this site is dispensable for G4 unfolding unless encountering very stable G4s (Wu and Spies, 2016). In addition, FANCJ disrupts G4 DNA stepwise and repetitively (Wu and Spies, 2016). The RSM domain of DHX36 is special for parallel G4 recognition (Figures 4A–C). Interestingly, this domain is dispensable for G4 DNA stabilization in the absence of ATP. However, RSM is essential for G4 unfolding (You et al., 2017). RHAU can also resolve RNA G4 stepwise and repetitively in a ATP-dependent manner (Tippana et al., 2019), and can unfold very stable RNA G4 formed by C9orf72 repeat RNA (Liu H. et al., 2021).

The kinetics of helicase unwinding activities is very important for us to understand their properties, and we attempted to comprehensively summarize and compare their kinetics. However, the G4 unwinding activities of helicases are ATP dependent and can be influenced by experimental conditions *in vitro*. Therefore, it is difficult for us to compare their unwinding kinetics reported by different studies. Fortunately, we can get partial information. First, different helicases have the ability to uniquely unwind the same G4 structure; for example, MOV10L1 is more efficient than that of MOV10 (Zhang et al., 2019). FANCJ is more efficient for unimolecular G4 DNA unwinding than DDX11 or XPD (Bharti et al., 2013). Second, the same helicases unwind the different G4 structures with different abilities; for example, ScPIF1 prefers unfolding antiparallel G4 DNA structures to parallel structures having similar thermal stability (Wang et al., 2018). DHX36 selectively recognizes and binds parallel G4s (Tippana et al., 2014). Finally, double-stranded DNA (dsDNA) can compete with G4 recognition of RecQ family helicases, because both of their recognition are dependent on the same RQC domain. However, for some helicases, such as ScPIF1, the unwinding mechanisms of G4 and dsDNA are different, where monomeric ScPIF1 can robustly unfold G4 DNA, but cannot efficiently unwind dsDNA, whose efficient unwinding need ScPIF1 dimer (Zhang et al., 2016).

**FIGURE 5:**
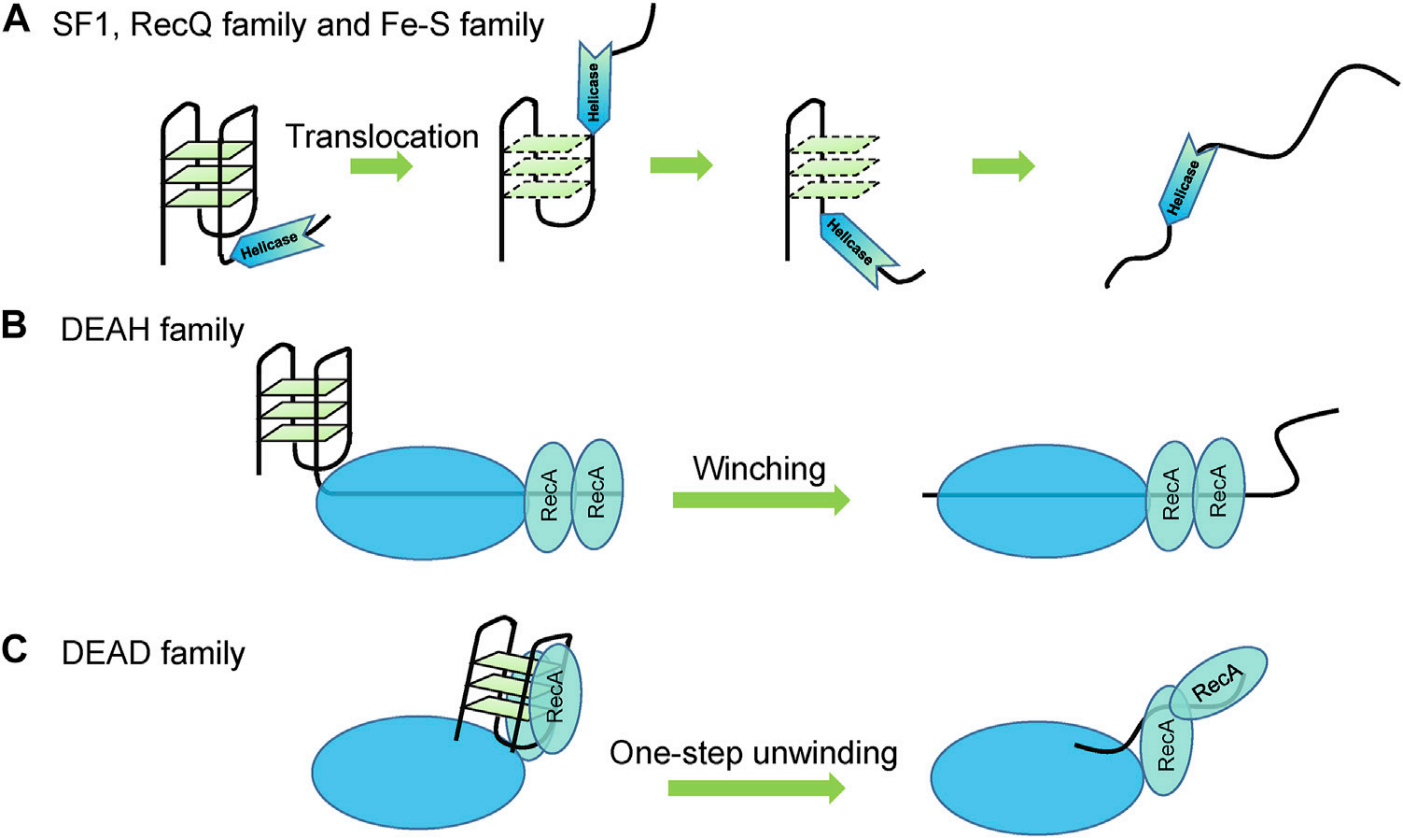
Proposed G4 unwinding mechanisms. **(A)** Translocation of SF1, RecQ family, and Fe-S family helicases. **(B)** Winching of DEAH family helicases. **(C)** Non-processive and one-step unwinding of DEAD family helicases.

Although different helicases exhibit different G4 unwinding behaviors, all the reported G4 unwinding helicases share the same duplex DNA unwinding mechanisms such as translocation of SF1, RecQ family, Fe-S family (Singleton et al., 2007) (Figure 5A), and winching of DEAH family (Gilman et al., 2017) (Figure 5B). In addition, the ssDNA tail is indispensable for helicase loading to initiate unwinding. Furthermore, both G4 DNA and RNA structures were unfolded step-by-step with stable intermediates such as G-triplexes and G-hairpins, just as their proposed folding and unfolding pathways (Wu et al., 2015; Tippana et al., 2019; Zhang et al., 2019b). The unwinding mechanisms of DEAD family helicases have not been revealed. Even DEAD and DEAH helicases are evolutionarily conserved but exhibit distinct RNA unwinding mechanisms (Gilman et al., 2017); thus, their G4 unfolding characteristics should also be different. Repetitive unfolding is also often detected, and seems to be a common mechanism of G4 unwinding helicases (Tippana et al., 2016). DEAD family helicases are typically poorly processive or non-processive (Cordin et al., 2006); thus, they may resolve the whole structure in one step (Figure 5C), just as the unwinding of duplex substrates (Sun et al., 2012; Sun et al., 2014).

Distinct from helicases, some specialized non-helicase proteins can resolve G4 structures (Table 2), as they do not require the energy provided by ATP hydrolysis and unfold G4 from passive binding. RPA is a single-stranded DNA binding protein that contains three subunits including 70-kDa (RPA1), 32-kDa (RPA2), and 14-kDa (RPA3). It can tightly bind ssDNA through its multiple oligonucleotide/oligosaccharide-binding (OB) fold domains and disrupt G4 DNA with 5′-3′ direction (Ray et al., 2013; Safa et al., 2016). This G4 unfolding activity is involved in replication (Lee et al., 2021) and telomere maintenance (Ray et al., 2014). As the key replication factor, RPA also contacts with many replication helicases including G4 unwinding helicases (Table 2). Protection of telomeres 1 (POT1) is crucial for telomere integrity by binding the 3′ end overhang to prevent the G4 formation, using its OB fold domains (Hwang et al., 2012; Ray et al., 2014). Recently, POT1 was reported to specially unfold telomeric G4 structures regardless of what the topology is, but not other G4 structures (Chaires et al., 2020), consistent with its cellular functions. CTC1-STN1-TEN1 (CST1), an RPA-like single-strand binding complex, can unfold G4 DNA *in vitro* (Bhattacharjee et al., 2017) and prevent G4 DNA accumulation during replication (Zhang M. et al., 2019). Cellular nucleic acid binding protein (CNBP), also known as zinc finger protein 9, unfold G4 DNA to control transcription (Michelotti et al., 1995; David et al., 2019), and G4 RNA to promote translation (Benhalevy et al., 2017). G-rich sequence factor 1 (GRSF1) can melt G4 RNA in noncoding RNA using a quasi-RNA-recognition motif (qRRM) to promote their degradation in mitochondria (Pietras et al., 2018). hnRNP family proteins are closely related to human health and diseases (Geuens et al., 2016). A large number of them contain G4 RNA binding domains, such as RRM and Arg-Gly-Gly (RGG) domains to specially recognize both G4 RNA and G4 DNA (Khateb et al., 2004; Wang et al., 2012; Ghosh and Singh, 2018; Izumi and Funa, 2019; Kharel et al., 2020b). Between them, hnRNP A1, its derivative UP1, and hnRNP H/F are among the most studied. By destabilizing G4 DNA and RNA and keeping ssDNA/RNA in an unfolded form (Clarke et al., 2021), hnRNP A1 and UP1 can modulate replication (Fukuda et al., 2002), transcription (Paramasivam et al., 2009), and telomere maintenance (Krüger et al., 2010; Ghosh and Singh, 2018). hnRNP H/F specifically binds unfolded G4 RNA to prevent G4 structure formation (Samatanga et al., 2012; Herviou et al., 2020). Through contact with DHX36, they can modulate translation (Herviou et al., 2020), and are recruited to RNA splicing sites to regulate RNA G4-mediated alternative splicing (Conlon et al., 2016; Huang et al., 2017).

**TABLE 2:** Non-helicase proteins have been proposed to bind and destabilize G4s.

Name	DNA or RNA	G4-modulated functions	Binding helicase
RPA	DNA G4 Ray et al. (2013), Safa et al. (2016)	Replication Lee et al. (2021) and telomere maintenance Ray et al. (2014)	PIF1 Maestroni et al. (2020), DNA2 Bae et al. (2003), BLM Brosh et al. (2000), WRN Lee et al. (2018), RECQ5β Garcia et al. (2004), FANCJ Lee et al. (2021), and DDX11 Farina et al. (2008)
TOP1	DNA G4 Chaires et al. (2020)	Telomere maintenance Ray et al. (2014)	BLM and WRN Opresko et al. (2005)
CST1	DNA G4 Bhattacharjee et al. (2017), Zhang et al. (2019a)	Replication and telomere maintenance Zhang et al. (2019a)	
CNBP	DNA G4 Benhalevy et al. (2017) and RNA G4 Michelotti et al. (1995), David et al. (2019)	Transcription Michelotti et al. (1995), David et al. (2019) and translation Benhalevy et al. (2017)	
GRSF1	RNA G4 Pietras et al. (2018)	RNA degradation in mitochondria Pietras et al. (2018)	
hnRNPA1	DNA G4 and RNA G4 Clarke et al. (2021)	Replication Fukuda et al. (2002), transcription Paramasivam et al. (2009), and telomere maintenance Krüger et al. (2010), Ghosh and Singh, (2018)	
hnRNPA2	DNA G4 and RNA G4 Khateb et al. (2004)	Translation Khateb et al. (2007)	
hnPNPA2*	DNA G4 Wang et al. (2012)	Telomere maintenance Wang et al. (2012)	
hnRNP H/F	RNA G4 Samatanga et al. (2012), Herviou et al. (2020)	Translation Herviou et al. (2020) and splicing Conlon et al. (2016), Huang et al. (2017)	DDX5 Dardenne et al. (2014), DDX17 Dardenne et al. (2014) and DHX36 Herviou et al. (2020)

Non-helicase proteins can also contact with G4 winding helicases. However, their cooperation with helicases has not been well studied. Recently, Hou’s group used smFRET to study the relationship between RPA, PIF1, and BLM (Wang et al., 2021). They found that both RPA and helicases were need for stable G4 DNA unfolding, and RPA played a complementary role for G4 unfolding by helicases (Wang et al., 2021).

## 5 Evolutionary Perspective

As determined by sequencing, G4 DNA exist in multiple species (Marsico et al., 2019). However, the evolution of G4s remains understudied. In recent years, some comparative analyses have been published. In bacteria, RNA G4s tend to be depleted (Guo and Bartel, 2016). In contrast, G4s in fungi are evolutionarily conserved (Capra et al., 2010). Additionally, G4s are significantly enriched in transcriptional regulatory regions of mammals (Zhao et al., 2007). Most recently, G4s from 37 evolutionarily representative species have been analyzed comprehensively (Wu et al., 2021). The authors found that the number, length, and density of G4s increased evolutionarily. Furthermore, G4s with short-loops are conserved in most species (Wu et al., 2021). It is possible that as evolution progresses, organisms become more complex, thus requiring more dimensions to regulate increasingly complex life processes, with G4 providing the possibility for finer adjustments within living organisms. By this logic, G4s are selected during evolution. In terms of helicases, it seems that the G4 unwinding abilities of members within the same family are conserved. For example, most members of the RecQ and PIF1 family helicases from bacteria to humans can efficiently unfold G4s (Wu and Maizels, 2001; Huber et al., 2002; Sanders, 2010; Hou et al., 2015; Liu et al., 2015; Wu et al., 2015; Wu et al., 2017), although there are some subtle differences between them. This situation is evidently due to evolutionary pressures leading to each domain being conserved, ultimately determining the unwinding ability of each helicase.

## 6 Future Prospects

Above, we have summarized the cellular functions, research methods, and mechanisms of G4 unwinding helicases. Although important progress has been made, there are still many important questions that need to be answered.

G4 DNA in mitochondria need to be unwound to maintain mitochondrial genome stability (Bharti et al., 2014; Micol et al., 2019; Dahal et al., 2021), and G4 RNAs also participate in mitochondrial RNA metabolism (Pietras et al., 2018). There are five helicases located in human mitochondria, including TWINKLE, SUV3, PIF1, DNA2, and RECQ4 (Peter and Falkenberg, 2020). Among them, the replicative TWINKLE helicase was unable to resolve G4 DNA structures (Bharti et al., 2014). PIF1, DNA2, and RECQ4 are G4 DNA unwinding helicases (Table 1), and their deficiencies are associated with mtDNA replication and stability (Peter and Falkenberg, 2020), while the G4-associated function details still require investigation. SUV3 RNA and DNA helicase may join in mitochondrial RNA G4 unwinding (Pietras et al., 2018); however, direct evidence that SUV3 unwinds G4s is lacking.

In terms of helicases, the RNA G4 unwinding mechanisms of most RNA helicases have not been studied. Importantly, the DEAD family helicases are very likely to disrupt the G4 structures in one step, just as they remodel the double strand (Sun et al., 2012; Sun et al., 2014). In addition, there are no DExH RNA helicases identified as G4 unwinding helicases. Interestingly, many helicases (DEAD and DEAH families) traditionally classified as RNA helicases can effectively bind and resolve the DNA G4 structures *in vivo* and *in vitro* (Table 1). However, most of their DNA G4-related cellular functions remain unclear. Recently, Yang’s lab identified that DDX5 unfolds the Myc promoter G4 to modulate its expression. This provides an example of an RNA helicase functioning by regulating G4 DNA (Wu et al., 2019). Additionally, it is worth studying whether the same helicase opens DNA G4 and RNA G4 using the same mechanism (Chakraborty and Grosse, 2011; Chen et al., 2015). Although most G4 DNA helicases have been well studied, the mechanism by which different helicases can simultaneously resolve the same G4 remains unclear. For example, BLM, WRN, and FANCJ were shown to cooperate in cells (von Kobbe et al., 2002; Sarkies et al., 2012), but it is still unclear whether there is synergy between them. Furthermore, the cooperations between non-helicase proteins (Table 2) and their interacting helicases are also worth investigating.

In terms of G4s in the human genome, most G4s are noncanonical structures (Chambers et al., 2015), such as bulged G4s (Zhang Y. et al., 2021), long looped G4s (Agrawal et al., 2014), and vacancy G4s (Li et al., 2015; Wang et al., 2020). However, there is little research on the interaction of G4 helicases with these structures. Additionally, DNA: RNA hybrid G4s also exist in cells and play an important role in cellular processes, including telomere maintenance and transcription (Xu et al., 2012; Xiao et al., 2014; Zhang et al., 2014). There are some G4 unwinding helicases reported to have the ability to unwind DNA: RNA hybrid duplex substrates, such as PIF1 (Boulé and Zakian, 2007), BLM (Chang et al., 2017), and DDX1 (Li et al., 2016). Whether they can resolve hybrid G4s and how they unfold hybrid G4s need to be further studied.

It should be noted that monitoring G4 unwinding processes in living cells is the ultimate goal of scientists. Taking advantage of the development of G4 fluorescent probes, researchers can observe the dynamics of G4s in real time in live cells. Ligands include, but are not limited to: IMT (Zhang et al., 2018) and DAOTA-M2 (Summers et al., 2021) for G4 DNA, QUMA-1 (Chen et al., 2018c) for G4 RNA, and N-TASQ for both G4 DNA and RNA (Laguerre et al., 2016). Recently, SiR-PyPDS has been found to allow individual G4 DNA detection in real time and at the single-molecule level (Di Antonio et al., 2020). It can be optimistically expected that such tools will make it possible to study the unwinding of G4 in cells.
