# Corrigendum: The Cellular Functions and Molecular Mechanisms of G-Quadruplex Unwinding Helicases in Humans

**DOI:** 10.3389/fmolb.2021.833430

**Published:** 2022-01-21

**Authors:** Yang Liu, Xinting Zhu, Kejia Wang, Bo Zhang, Shuyi Qiu

**Affiliations:** ^1^ Key Laboratory of Plant Resource Conservation and Germplasm Innovation in Mountainous Region (Ministry of Education), Collaborative Innovation Center for Mountain Ecology and Agro-Bioengineering (CICMEAB), College of Life Sciences/Institute of Agro-bioengineering, Guizhou University, Guiyang, China; ^2^ College of Basic Medicine, Zunyi Medical University, Zunyi, China; ^3^ The Key Laboratory of Fermentation Engineering and Biological Pharmacy of Guizhou Province, Guizhou University, Guiyang, China; ^4^ School of Liquor and Food Engineering, Guizhou University, Guiyang, China

**Keywords:** G-quadruplex, helicase, genetic disease, age-related diseases, cancer

In the published article, there was an error in **Affiliation** “1.” Instead of “College of Life Sciences, Guizhou University, Guiyang, China,” it should be “Key laboratory of Plant Resource Conservation and Germplasm Innovation in Mountainous Region (Ministry of Education), Collaborative Innovation Center for Mountain Ecology and Agro-Bioengineering (CICMEAB), College of Life Sciences/Institute of Agro-bioengineering, Guizhou University, Guiyang, Guizhou Province, China.”

The authors apologize for this error and state that this does not change the scientific conclusions of the article in any way. The original article has been updated.

